# Understanding Biomarker Discordance: MRI and PET Phenotypes Shed Light on Alzheimer’s Pathology

**DOI:** 10.1002/alz.095708

**Published:** 2025-01-09

**Authors:** Tamil Iniyan Gunasekaran, Annie J. Lee, Patrick J. Lao, Jose Gutierrez, Jennifer J. Manly, Yian Gu, Adam M. Brickman, Lawrence S. Honig, Badri N. Vardarajan, Richard Mayeux

**Affiliations:** ^1^ The Gertrude H. Sergievsky Center, College of Physicians and Surgeons, Columbia University, New York, NY USA; ^2^ Department of Neurology, The New York Presbyterian Hospital, New York, NY USA; ^3^ Taub Institute for Research on Alzheimer’s Disease and the Aging Brain, Columbia University, New York, NY USA; ^4^ Taub Institute for Research on Alzheimer’s Disease and the Aging Brain, Vagelos College of Physicians and Surgeons, Columbia University, New York, NY USA; ^5^ Department of Neurology, Vagelos College of Physicians and Surgeons, Columbia University, and the New York Presbyterian Hospital, New York, NY USA; ^6^ Department of Neurology, College of Physicians and Surgeons, Columbia University, New York, NY USA; ^7^ Department of Neurology, Columbia University Medical Center, New York, NY USA; ^8^ Gertrude H. Sergievsky Center, Vagelos College of Physicians & Surgeons, Columbia University, New York, NY USA

## Abstract

**Background:**

Plasma biomarkers for Alzheimer’s disease (AD) are indicators of neuropathological changes. Biomarker cutoffs may not consistently indicate the presence or absence of a pathology. Discrepant biomarker levels are observed in some cognitively normal subjects and patients with clinically diagnosed AD, with some asymptomatic individuals having biomarker evidence of AD pathophysiology and some symptomatic individuals without evidence of AD pathophysiology. The purpose of this study is to understand the discordant biomarker levels among CN and AD using phenotypes from MRI and PET images.

**Method:**

We measured phosphorylated tau 181 (P‐tau181) in plasma from 685 individuals in the Washington Heights Inwood Columbia Aging Project (WHICAP; Mean age = 76.52; women = 64.59%; non‐Hispanic Whites = 29.61%, African Americans = 30.62%; Caribbean Hispanics = 37.30%) and characterized them as biomarker positive based on a ptau181 cutoff of 2.648 pg/mL: 556 cognitively normal (CN, 241 biomarker+), 59 mild cognitively impaired (MCI, 26 biomarker+), and 70 had clinical AD (40 biomarker+). We used MRI (n = 685) and [18F] Florbetaben (FBB) Aβ PET in 167 (24.2%) individuals, and [18F]‐MK‐6420 tau PET 23 (3.36%) individuals) to compare white matter hyperintensity volume (WMH), hippocampus volume, number of silent brain infarcts (SBI), Aβ standard uptake value ratios (SUVR), and tau SUVR among the six biomarker groups (CN‐, CN+, MCI‐, MCI+, AD‐, AD+) with ANCOVA model adjusting for age, sex, and ethnicity.

**Result:**

The AD+ group had larger WMH volume (*P* = 2.81×10^−26^), smaller hippocampus volume (*P* = 5.34×10^−04^), higher Aβ SUVR (*P* = 2.26×10^−03^) and tau SUVR (*P* = 8.11×10^−04^) compared to the CN‐. The AD‐ group had larger WMH volumes (*P* = 1.96×10^−04^) and smaller hippocampal volume (*P* = 0.039) to CN‐. When compared to the AD‐ group, the AD+ group had larger WMH volume (*P* = 4.34×10^−03^). The CN+ group had a larger WMH volume (*P* = 4.17×10^−03^) and smaller hippocampal volume (*P* = 4.13×10^−05^) than CN‐ group. Both MCI+ (*P* = 0.048) and MCI‐ (*P* = 0.022) groups showed smaller hippocampal volume compared with the CN‐ group. Except for hippocampal volume, no other phenotypes demonstrated statistical significance in the MCI+ and MCI‐ groups.

**Conclusion:**

MRI and PET phenotypes exhibited a strong association with pathophysiological changes in the AD+ group. Specifically, MRI phenotypes such as WMH volume and hippocampal volume could be instrumental in characterizing AD pathophysiology, even with discrepancies in biomarker profiles.

